# Seed Germination and Seedling Growth of *Dendrocalumus brandisii in vitro*, and the Inhibitory Mechanism of Colchicine

**DOI:** 10.3389/fpls.2021.784581

**Published:** 2021-12-23

**Authors:** Zhuo Lv, Fangwei Zhu, Diankun Jin, Yufang Wu, Shuguang Wang

**Affiliations:** ^1^Key Laboratory for Sympodial Bamboo Research, Faculty of Life Sciences, Southwest Forestry University, Kunming, China; ^2^Science and Technology Innovation Team of National Forestry and Grassland Administration, Southwest Forestry University, Kunming, China

**Keywords:** bamboo seed, germination, seedling growth, sugar metabolism, inhibitory mechanism, colchicine

## Abstract

Bamboos seldom bloom and almost no seeds could be harvested, and, hence, few works are focused on germination physiology. Systematic research on the physiological effects of colchicine on germination and seedling growth of bamboo seeds is lacking. In this study, we finely recorded seed germination and seedling growth of *Dendrocalamus brandisii* in media supplemented with different colchicine concentrations. Physiological effects and mechanisms of colchicine were analyzed. The results showed that *D. brandisii* seeds were non-dormant, and seed lots achieved their highest germination rates on the 4th day and finished the whole germination period after 21 days. Colchicine inhibited seed germination and seedling growth but did not change its germination pattern. Seed germination and seedling growth decreased constantly with colchicine concentration. Colchicine showed more negative effects on seedling growth than on seed germination and root growth. High concentrations of colchicine retarded the development of plumules and even caused their aberrant development. Under tissue culture conditions, seed germination, and seedling growth relied mainly on the endogenous starch and soluble sugar degradation, in which α-amylase, STP, and SUSY played the key role. Colchicine inhibited seed germination and seedling growth by suppressing the α-amylase, STP, and SUSY activities. Colchicine showed more negative effects on sucrose degradation than on starch degradation during seed germination and seedling growth. This study provides new basic information on the seedling physiology for the genetic breeding of bamboo plants.

## Introduction

Bamboos seldom bloom, and their vegetative growth intervals are reported as 50–60 years (Lin et al., 2010). Due to the long intervals of vegetative growth, almost no research has been focused on the genetic breeding of bamboo plants. In addition, seed productions are very low. In some bamboo species, such as *Dendrocalamus giganteus*, no seeds have ever been collected (Wang et al., 2015). This further limits the genetic breeding of bamboo plants.

Colchicine is an alkaloid contained in the seed or bulb of *Colchicum autumnale* belonging to the family of Liliaceae (Kwon et al., 2016). Colchicine is used in breeding and biological research to induce polyploidy and in tubulin-binding assays as a positive control (Trease and Evans, 1983). It interferes with chromosome movement to poles and microtubules formation in the middle stage of cell division and induces polyploidization (Hadlaczky et al., 1983). The effect of colchicine for *in vitro* chromosome doubling is related to its concentration, method, and duration of treatment (Dhooghe et al., 2011; Aina et al., 2012; Moghbel et al., 2015; Kwon et al., 2016).

Bamboo species in *Dendrocalamus* are usually considered hexaploids (2n = 70–72) (Chen et al., 2003). However, Li et al. (2011) reported that dodecaploidy could be induced during the pollen culture of *D. latiflorus*. This work made us realize that the chromosome number in cells of sympodial bamboos might be doubled again. Hence, we attempted to induce the dodecaploidy of *D. brandisii* with the use of seed culture supplemented with different concentrations of colchicine. After 3 years of works on seed culture, no apparent seedlings of dodecaploidy were obtained according to the appearance and morphological observations, but we noticed that colchicine significantly retarded seed germination and the subsequent height of growing seedlings.

To the best of our knowledge, most works have focused on polyploid breeding, but few are concerned about the physiological effects of colchicine on seed germination and seedling growth and the related mechanism. As a kind of starchy seeds, starch, and sucrose metabolism and seed germination are strongly linked. Therefore, it is essential to analyze dynamic changes in sugar metabolism during the germination process of *D. brandisii* seeds. In this paper, we recorded seed germination characteristics, determined the related sugar metabolism, and analyzed the physiological effects of colchicine on seed germination and the subsequent growth to reveal the physiological mechanism of colchicine that retarded the germination and seedling growth and supply new basic information on the seedling physiology for breeding of bamboo plants.

## Materials and Methods

### Plant Materials

Seeds of *D. brandisii* were collected from a flowering bamboo forest in Jinggu County (102°761 E and 25°061 N) of Yunnan province, China. The length and the width of 150 seeds with lemma and without lemma were separately determined by using a vernier caliper (accuracy 0.01 mm). The thousand-seed weight was also determined three times.

After de-husking, the healthy-looking seeds were selected and sterilized in 70% ethanol for 30 s and then in 0.1% HgCl_2_ for 10 min, followed by washing five times in sterile deionized water. After sterilization, the seeds were inoculated on a basic Murashige and Skoog (1962) (MS) medium in culture tubes, containing 2.5% sucrose, 0.8% agar, 0.5 mg/L BA, and different concentrations of colchicine (0.00, 0.05, 0.10, 0.20, 0.30, 0.40, 0.50, and 0.60%). The seeds were cultured at 25°C under a 16-h light/8-h dark photoperiod with the photosynthetic photon flux density (PPFD) of 27-μmol m^−2^ s^−1^ light. The colchicine solution was filter-sterilized. All data of seed germination, seedling growth, and the related physiological experiments were recorded and performed in one culture generation. Each treatment included three replicates with 50 culture tubes in each replicate.

### Seed Germination and Seedling Growth

Germination data of *D. brandisii* seeds were recorded carefully, including germination rate, germination potential, death rate, and seedling survival rate. The germination rates of seeds in media supplemented with different concentrations of colchicine were recorded every day, until the 21st day, when the seeds did not germinate anymore. Starting on the 2nd day of observation on culture tubes, the germinated seeds based on a radicle length of 0.2 cm were used as the germination standard (Li et al., 2021). The germination rate reached its highest values on the 4th day, and then the germination potential was calculated based on the number of germinated seeds by Day 4.

Germination rates (%) = (Total number of seeds that germinated/Total number of tested seeds) × 100.
Germination potential (%) = (Number of germinated seeds by Day 4/Total number of tested seeds) × 100.
Death rates (%) = (Number of dead seeds/Total number of tested seeds) × 100.
Seedling survival rates (%) = (Number of survival seedlings by Day 26/Total number of tested seeds) × 100.

In addition, the height, culm diameter, and weight of seedlings under different concentrations of colchicine were also determined after 26 days to analyze the influence of colchicine on seedling growth.

### The Anatomical Structure of Seeds Treated With Colchicine

The germination process of *D. brandisii* was divided into four stages, i.e., ungerminated stage, radicle emerging stage, plumule emerging stage, and seedling stage. The seeds in ungerminated and radicle emerging stages on normal culture media and on those supplemented with 0.6% colchicine were gathered and fixed in FAA to analyze the influence of colchicine on anatomical structure. After the fixation, the seed specimens were cut into 10-μm thick paraffin sections by using a rotary microtome (Leica) and stained with safranin and fast green. Sections were observed under a fluorescent microscope (Nikon E400, Tokyo, Japan).

### Endogenous Soluble Sugars and Starch Contents Determination

Total endogenous soluble sugars were extracted according to the method of Glassop et al. (2007). About 2 g of specimens were grounded in liquid nitrogen and extracted with 10 ml of deionized water at 70°C. The homogenates were centrifuged at 12,000 g for 20 min, and the supernatants were collected for soluble sugar content determination. The sediments were stored at −20°C for starch content determination. The total endogenous soluble sugar and starch contents were determined according to the phenol-sulfuric acid method (Dubois et al., 1956). The measurement of each sample was repeated three times.

### Activities of Sucrose-Metabolizing Enzymes

All related enzymes were determined for their activities after seed hydration and at other germination stages. For an invertase assay, 1-g seed sample was homogenized in liquid nitrogen and added into centrifuge tubes containing a 1-ml extraction buffer consisting of 50-mM KPO_4_ (pH 7.5), 5-mM MgCl_2_, and 1-mM EDTA (Lingle and Dunlap, 1987). The tubes were centrifuged in a refrigerated centrifuge (Xiangyi) at 12,000 g and 4°C for 30 min. The supernatants were collected in dialysis tubes (10 KD cutoff, Sigma-Aldrich) and dialyzed against 1 l of 10-mM KPO_4_ (pH 7.5). The dialysis was carried out overnight at 4°C. The dialysate was used for soluble acid invertase (SAI) assays. The pellet was collected and washed three times with the extraction buffer and resuspended in the same buffer for the insoluble extracellular invertase (CWI) assay. The extracts were incubated within a 1-M sodium acetate buffer (pH 4.5) and 100-mM sucrose at 37°C for 30 min. The reactions were stopped by adding 30 μl of 2.5-M Tris and boiled at 100°C for 5 min. The SAI and CWI activities were expressed in μmol NADH per min per g fresh tissue.

To determine sucrose synthase (SUSY), 1-g seed samples were homogenized in liquid nitrogen and extracted in 1 ml of an extraction buffer containing a 50-mM hydroxyethyl piperazine ethane sulfonic acid buffer (HEPES)/NaOH (pH 7.5), 7.5-mM MgCl_2_, and 1-mM EDTA, 2% (w/v) PEG 8,000, 2% (w/v) polyvinylpyrrolidone (PVP), and 5-mM dithiothreitol (DTT; Hoffmann-Thoma et al., 1996). The extracts were centrifuged as described above. The cleavage activity of SUSYC was determined according to Lowell et al. (1989). The reaction was performed at 37°C for 30 min in the extraction buffer containing an 80-mM MES buffer (pH 5.5), 5-mM NaF, 100-mM sucrose, and 5-mM UDP. The activities were expressed in μmol NADH per min per g fresh tissue.

### Activities of Starch-Metabolizing Enzymes

For the activity determination of starch-metabolizing enzymes, the crude enzyme was extracted according to Nakamura et al. (1989) and Sergeeva et al. (2012). All the procedures were carried out at 0°C. Seed samples (1 g) were homogenized in liquid nitrogen and added to centrifuge tubes, containing a 1-ml extraction buffer containing a 100-mM HEPES buffer (pH 7.4); MgCl_2_, 5 mM; EDTA, 2 mM; 10% (v/v) glycerol;0.1% BSA; 5-mM DTT; and 2% (w/v) insoluble PVP, and then were centrifuged at 12,000 g and 4°C for 30 min. The supernatants were preserved at 4°C for the following activity determination. The remaining pellets were resuspended in 1 ml of an extraction buffer for the insoluble enzyme assay.

AGPase activities of seed samples at different germination stages were conducted in 1 ml of the following solution: a 100-mM HEPES buffer (pH 7.4), 1.2-mM ADPG, 3-mM PPi, 5-mM MgCl_2_, 4-mM DTT, and 100-ml enzyme extract (Nakamura et al., 1989). Reactions were performed at 37°C for 30 min and were ended in boiling water for 10 min. After centrifugation of 12,000 g for 10 min, 500 μl of the supernatants were mixed with 15 μl of 10-mM NAD. The activity was calculated in terms of μmol NADH per min per g fresh tissue by measuring the increase in absorbance at 340 nm after the addition of 1 μl of P-glucomutase (0.4 U) and 1 μl of glucose-6-phosphate dehydrogenase (0.35 U).

Soluble starch synthase (SSS) and granule-bound starch synthase (GBSS) activities were determined according to Nakamura et al. (1989) with some modifications. The assay was conducted in a 50-mM HEPES buffer (pH 7.4), 1.6-mM ADPG, 0.7-mg amylopectin, 15-mM DTT, and enzyme preparation in a reaction mixture of 280 μl. It was terminated in boiling water for 10 min after the 30-min reaction at 37°C. Then, 100 μl of a solution of a 50-mM HEPES buffer (pH 7.4), 4-mM PEP, 200-mM KCl, 10-mM MgCl2, and 1.2 U pyruvatekinase was added and incubated for 30 min at 37°C before being terminated by boiling for 10 min. After centrifugation of 12,000 g for 10 min, 300 μl of the supernatant were mixed with a 50-mM HEPES buffer (pH 7.4), 10-mM glucose, 20-mM MgCl_2_, and 2-mM NAD. The activities were measured as the increase in absorbance at 340 nm after the addition of 1 μl each of hexokinase (1.4 U) and glucose-6-phosphate dehydrogenase (0.35 U). The GBSS activity was measured by using the same method, and the suspended pellet was mixed with the reaction buffer instead of the supernatant. The activities were also expressed in μmol NADH per min per g fresh tissue.

Starch phosphorylase (STP) was assayed in a final volume of a 1-ml medium containing a 50-mM HEPES buffer (pH 7.), 0.4% soluble starch, 0.4-mM NAD, 1-U phosphoglucomutase, 1-U glucose-6-phosphatedehydrogenase, 15-mM glucose-1,6-bisphosphate, and 10-mM Na_3_PO_4_, and 50-μl enzyme extract (Appeldoorn et al., 1999). The reaction proceeded for 30 min at 37°C; after which, it was terminated by boiling for 10 min. The activities were measured as the increase in absorbance at 340 nm and expressed in μmol NADH per min per g fresh tissue.

α- and β-amylase activities were measured according to the method of Hesam et al. (2015) and Wang and Huang (2015). The enzymatic activity was determined using starch as the substrate (1% w/v in a 100-mM citrate-phosphate buffer at pH 6). The hydrolysis of starch at 50°C was monitored by the determination of the reducing sugar using the dinitrosalicylic acid method at 540 nm. A standard curve was prepared with maltose. The activities were expressed in mg maltose per min per g fresh tissue.

### Statistical Analysis

The data presented were the means of three independent experiments with 3–5 replicates for each experiment (means ± SD). For the comparisons of all indexes obtained during different seed germination stages, the significance was statistically evaluated by using the least significant difference (LSD) test. While, for the comparisons of those indexes obtained from seeds or seedlings inoculated in the two different media, the two-way ANOVA was used. Differences were considered significant at *p* < 0.05.

## Results

### Morphological and Anatomical Observations on the Germination of *D. brandisii* Seeds

The seeds of *D. barndisii* were nut-like caryopsis with glumes outside. The glumes could be divided into palea and lemma, and the lemma was easy to fall off. The shape of the seed was nearly spherical with an obvious longitudinal groove on its abdomen, and the embryo was located at the base of the caryopsis. The length of the seeds was 5.45 ± 0.04 mm, the width was 2.93 ± 0.03 mm, and the thousand-grain weight was 28.19 ± 0.83 g.

**Figure 1:**
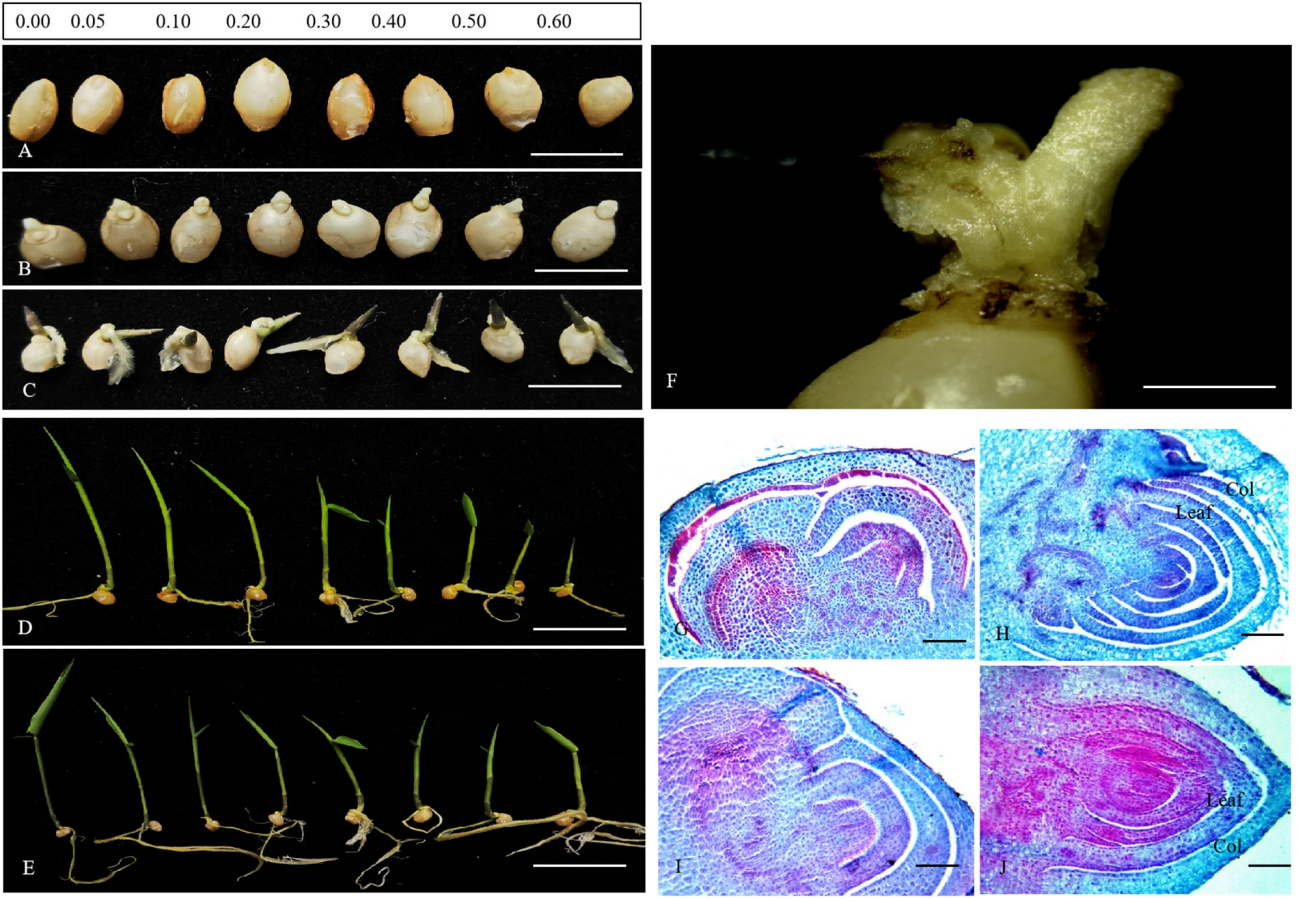
The morphological and anatomical observation on the germination of *D. brandisii* seeds. **(A)** The new inoculated seeds in the media supplemented with different concentrations of colchicine, which were in the ungerminated stage. Bar = 5 mm. **(B)** Seven days after inoculation, showing the seeds in the radicle-emerging stage. Bar = 5 mm. **(C)** Ten days after inoculation, showing the seeds in the plumule-emerging stage. Bar = 5 mm. **(D)** 16 days after inoculation, showing the seedling stage. Bar = 25 μm. **(E)** Twenty-six days after inoculation, showing the seedling stage. Bar = 25 μm. **(F)** Abnormal germination of bamboo seeds. Bar = 1 mm. **(G,H)** Anatomical characteristics of seeds in the media supplemented with colchicine. **(G)** Seeds at the germination initiation stage. Bar = 50 μm. **(H)** The seed plumule stage. Bar = 50 μm. **(I,J)** Anatomical characteristics of seeds in the media without colchicine. **(I)** Seeds at the germination initiation stage. Bar = 50 μm. **(J)** The seed plumule stage. Bar = 50 μm.

According to the observations on seed germination of *D. brandisii* in media, we divided the germination process into four stages, i.e., ungerminated stage, radicle emerging stage, plumule emerging stage, and seedling stage (Figures 1A–D). The seeds of *D. brandisii* were non-dormant. Most seeds could germinate normally after being inoculated in the media supplemented with different concentrations of colchicine (Figures 1A–C). The radicle emerged first from the top of the seeds, followed by the emergency of the plumule (Figures 1B,C). No significant differences were observed in germination for most seeds inoculated in the media supplemented with colchicine as compared to those in the media without colchicine based on the morphological observations. While, at the seedling stage of 16 and 26 days after inoculation, it could be noticed that the seedling height constantly decreased with the increase of colchicine concentrations with the lowest seedling height in the media containing 0.60% colchicine (Figures 1D,E). However, there was no significant difference in root development for the seedlings treated with different concentrations of colchicine, which implies that colchicine did not inhibit root growth but inhibited seedling height growth significantly during the seedling stage of *D. brandisii*.

Additionally, some seeds showed abnormal germination phenomenon, most of which were inoculated in those media containing colchicine, especially for those in the media containing high colchicine concentrations (Figure 1F). The radicle and plumule could not be differentiated normally anymore. Only a small mass of undifferentiated tissue appeared at the top of the seed, and, finally, these seeds died. The higher the colchicine concentrations, the more seeds germinated abnormally. According to the anatomical observations, the plumule of seeds in the media containing 0.6% colchicine developed more slowly as compared to those in the media without colchicine during the germination stage (Figures 1G–J). In the media without colchicine, the developmental degree of plumule was higher with more young foliage leaves differentiated in the plumule, as compared to those inoculated in the media containing colchicine.

### Germination Status of *D. brandisii* Seeds in the Media Containing Different Colchicine Concentrations

**Figure 2:**
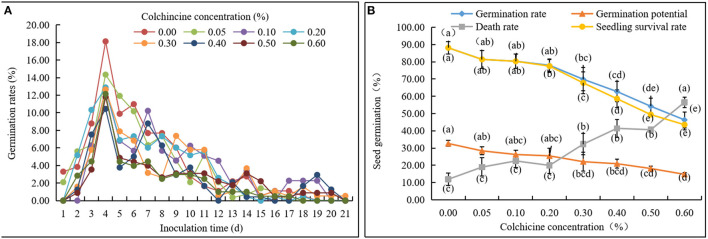
Germination status of *D. brandisii* seeds inoculated in media supplemented with different concentrations of colchicine. **(A)** Dynamical changes of seed germination rates with time. **(B)** Germination rates, germination potential, death rates, and seedling survival rates of seeds after 21 days of inoculation in media supplemented with different concentrations of colchicine. Different letters indicated significant difference at *P* < 0.05.

Seed germination rates in the media containing different colchicine concentrations were recorded every day (Figure 2A, Table 1). Noticeably, some seeds began to germinate on the 1st day after inoculation in all media. Germination rates achieved their highest values on the 4th day and then deceased constantly with time. The germination of all living seeds was finished in 21 days, except for those that had lost their viability and did not germinate anymore. Additionally, the germination rates of seeds inoculated in the media containing colchicine were lower than those of the seeds inoculated in the media without colchicine on the 4th day.

**Table 1:** Germination rates (%) of *Dendrocalamus brandisii* seeds inoculated in media containing different colchicine concentrations as a function of time (days).

**Colchicine concentration** **(%)**	**Inoculation time (day)**																				
	**1**	**2**	**3**	**4**	**5**	**6**	**7**	**8**	**9**	**10**	**11**	**12**	**13**	**14**	**15**	**16**	**17**	**18**	**19**	**20**	**21**
0.00	3.30 ± 2.16a	3.84 ± 1.70ab	8.79 ± 1.89a	18.14 ± 2.07a	9.89 ± 1.63ab	10.99 ± 2.05a	7.69 ± 1.25ab	7.69 ± 3.09a	6.04 ± 2.49a	2.75 ± 1.70a	1.65 ± 0.82a	1.10 ± 0.47b	2.20 ± 1.25a	2.75 ± 1.70a	0.55 ± 0.47ab	1.10 ± 0.47a	1.10 ± 0.47a	0.55 ± 0.47a	0.00 ± 0.00a	0.00 ± 0.00a	0.00 ± 0.00a
0.05	2.10 ± 1.63a	5.61 ± 0.47a	6.32 ± 1.41a	14.35 ± 1.70a	11.93 ± 0.94a	10.18 ± 2.49a	6.32 ± 0.82ab	7.37 ± 1.41a	4.56 ± 0.47a	2.10 ± 1.63a	5.26 ± 2.94a	2.10 ± 0.82ab	0.35 ± 0.47a	0.70 ± 0.94a	1.40 ± 0.47ab	1.05 ± 0.00a	0.00 ± 0.00a	0.00 ± 0.00a	0.00 ± 0.00a	0.00 ± 0.00a	0.00 ± 0.00a
0.10	0.00 ± 0.00a	0.00 ± 0.00b	6.25 ± 1.70a	12.66 ± 0.06a	6.81 ± 0.82bc	5.68 ± 0.47a	10.23 ± 1.41a	5.68 ± 0.94a	4.56 ± 1.25a	6.25 ± 1.25a	5.11 ± 1.41a	4.54 ± 0.94a	1.72 ± 0.82a	3.40 ± 2.83a	0.00 ± 0.00b	0.00 ± 0.00a	2.27 ± 1.89a	2.27 ± 1.89a	2.27 ± 1.25a	0.00 ± 0.00a	0.00 ± 0.00a
0.20	0.00 ± 0.00a	5.17 ± 0.82a	10.34 ± 0.82a	12.89 ± 2.25a	6.90 ± 1.25bc	7.33 ± 0.47a	6.03 ± 0.47ab	7.33 ± 0.47a	6.03 ± 0.47a	5.17 ± 1.41a	5.60 ± 0.94a	2.59 ± 0.82b	1.72 ± 0.47a	0.42 ± 0.00a	0.00 ± 0.00b	0.43 ± 0.47a	0.00 ± 0.00a	0.43 ± 0.47a	0.00 ± 0.00a	0.00 ± 0.00a	0.00 ± 0.00a
0.30	0.00 ± 0.00a	1.58 ± 1.41ab	5.79 ± 1.25a	12.66 ± 3.89a	7.89 ± 0.82bc	6.84 ± 1.70a	3.16 ± 1.41b	2.63 ± 1.70a	7.37 ± 0.47a	5.79 ± 2.62a	5.79 ± 1.70a	0.53 ± 0.47b	1.05 ± 0.47a	3.68 ± 0.47a	0.53 ± 0.47ab	1.05 ± 0.94a	0.53 ± 0.47a	1.58 ± 0.00a	0.53 ± 0.47a	0.53 ± 0.47a	0.53 ± 0.47a
0.40	0.00 ± 0.00a	1.25 ± 0.82ab	7.53 ± 2.45a	10.45 ± 1.13a	3.77 ± 1.41c	5.02 ± 2.16a	8.79 ± 2.94ab	6.28 ± 0.82a	2.93 ± 1.25a	3.77 ± 1.41a	1.67 ± 1.25a	0.00 ± 0.00ab	2.10 ± 1.25a	0.42 ± 0.47a	0.42 ± 0.47ab	0.00 ± 0.00a	0.00 ± 0.00a	1.67 ± 1.89a	2.93 ± 2.62a	1.26 ± 1.41a	0.00 ± 0.00a
0.50	0.00 ± 0.00a	0.88 ± 0.47ab	3.54 ± 0.47a	11.81 ± 0.00a	4.87 ± 0.47c	4.42 ± 1.25a	3.98 ± 0.00ab	2.65 ± 0.00a	3.10 ± 0.47a	3.10 ± 1.25a	3.10 ± 0.47a	2.21 ± 0.47ab	1.77 ± 0.47a	3.10 ± 0.47a	2.21 ± 0.47a	0.44 ± 0.47a	0.44 ± 0.47a	0.88 ± 0.47a	0.88 ± 0.94a	0.88 ± 0.47a	0.00 ± 0.00a
0.60	0.00 ± 0.00a	2.84 ± 0.47ab	4.46 ± 0.82a	12.15 ± 0.58a	4.45 ± 0.82c	3.96 ± 0.47a	4.46 ± 0.82ab	2.48 ± 0.47a	2.97 ± 0.82a	2.97 ± 0.82a	2.48 ± 0.47a	1.00 ± 0.47b	1.00 ± 0.47a	1.00 ± 0.47a	0.50 ± 0.47ab	0.50 ± 0.47a	0.50 ± 0.47a	0.00 ± 0.00a	0.00 ± 0.00a	0.00 ± 0.00a	0.00 ± 0.00a

*Means with the same letter in each line are not significantly different at P < 0.05*.

By comparing seed germination status in different media after 21 days, it was observed that the death rates of seeds increased constantly with the concentration of colchicine with the highest rate of 56.48 ± 2.95% at the colchicine concentration of 0.6%. At the same time, germination rates, seedling survival rates, and germination potential decreased constantly (Figure 2B, Table 2). However, their values between treatments of low colchicine concentrations (from 0.00 to 0.10%) were not significant, which indicated that the low concentrations of colchicine did not deteriorate the seed viability significantly.

**Table 2:** Germination status (%) of *Dendrocalamus brandisii* seeds treated with different colchicine concentrations after 21 days.

**Colchicine concentration**	**Germination percentage**	**Germination energy**	**Death rate**	**Survival rate**
0.00	88.09 ± 3.62a	18.14 ± 2.07a	11.91 ± 3.62c	88.09 ± 3.62a
0.05	81.37 ± 5.19ab	14.35 ± 1.70a	18.97 ± 5.32c	81.37 ± 5.19ab
0.10	80.28 ± 4.17ab	12.66 ± 0.06a	22.53 ± 3.73c	80.28 ± 4.17ab
0.20	77.93 ± 3.52ab	12.89 ± 2.25a	19.50 ± 4.61c	77.47 ± 3.73b
0.30	69.84 ± 6.72bc	12.66 ± 3.89a	32.26 ± 6.26b	67.74 ± 6.26c
0.40	62.73 ± 5.87cd	10.45 ± 1.13a	41.43 ± 5.19b	58.57 ± 5.19d
0.50	54.20 ± 4.78de	11.81 ± 0.00a	40.61 ± 0.43b	49.39 ± 0.00e
0.60	46.32 ± 4.57e	12.15 ± 0.58a	56.48 ± 2.95a	43.52 ± 2.95e

*Means with the same letter in each column are not significantly different at p < 0.05*.

It could also be noticed that seedling survival rates were the same as the germination rates at the colchicine concentrations of 0.00–0.10% but were lower at the concentrations of 0.10–0.60%. Meanwhile, the difference values between the germination rates and survival rates increased gradually with colchicine concentrations (Figure 2B, Table 2). These results implied that colchicine not only led to the increase of death rates of seeds but also decreased the germination rates and germination potential, especially for the high colchicine concentrations, which significantly decreased the later seedling survival rates.

### The Growing Status of *D. brandisii* Seedlings in the Media Containing Different Colchicine Concentrations

**Figure 3:**
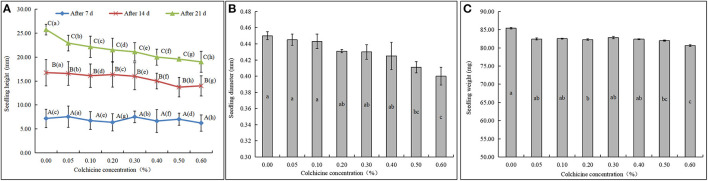
Growth status of *D. brandisii* seedlings in the media supplemented with different concentrations of colchicine. **(A)** The heights of seedlings cultured in the media were supplemented with different concentrations of colchicine after 7, 14, and 21 days. **(B)** The diameter of seedlings after 21 days. **(C)** The weight of seedlings after 21 days. Different lowercase letters indicated significant difference between colchicine concentrations and different uppercase letters indicated significant difference between different periods at *P* < 0.05.

The *D. brandisii* seedlings inoculated in the media containing different concentrations of colchicine showed different growing states (Figure 3). Seedling heights after the inoculation of 7, 14, and 21 days were recorded and compared, as well as the seedling diameter and weight after 21 days. After inoculation of 7 days, all seeds germinated to be seedlings, and no apparent rules could be drawn among the heights of seedlings in different media (Figure 3A). After 14 days, the height of seedlings inoculated in the media containing colchicine began to show lower values compared to that of the control. After 21 days, all the seedlings grew higher than those of 14 days, and the seedling height showed an apparent trend that constantly decreased with colchicine concentration. This result revealed that colchicine not only suppressed seed germination but also affected seedling height growth significantly.

In addition, the diameter and the weight of seedlings were also inhibited in the media containing colchicine, showing a decreasing trend with colchicine concentrations (Figures 3B,C). However, the decreasing trend of seedling height was not significant at the low colchicine concentrations from 0.00 to 0.10% but was significant at the high concentrations of 0.50 and 0.60%. Generally, higher concentrations of colchicine had higher suppressive effects on seedling growth of *D. brandisii*.

### Dynamical Changes of Endogenous Starch and Soluble Sugar Contents During Seed Germination and Seedling Growth

**Figure 4:**
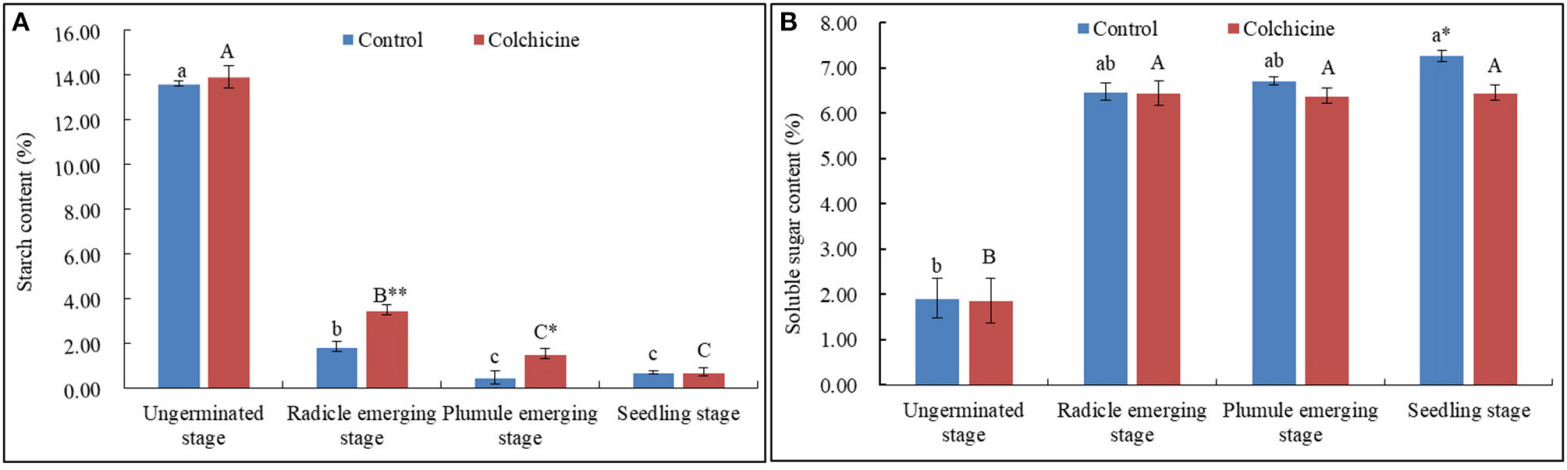
Dynamical changes of endogenous starch and soluble sugar contents in seeds with the germination process in the media supplemented with and without 0.6% colchicine. **(A)** Dynamical changes of the endogenous starch contents in seeds. **(B)** Dynamical changes of the endogenous soluble sugar contents in seeds. Different lowercase letters indicated significant difference between different developmental stages in the control, and different uppercase letters indicated significant difference between different developmental stages in the colchicine treatment at *P* < 0.05. *Indicated significant difference at *P* < 0.05, and **indicated significant difference at *P* < 0.01 between the control and colchicine treatment.

**Figure 5:**
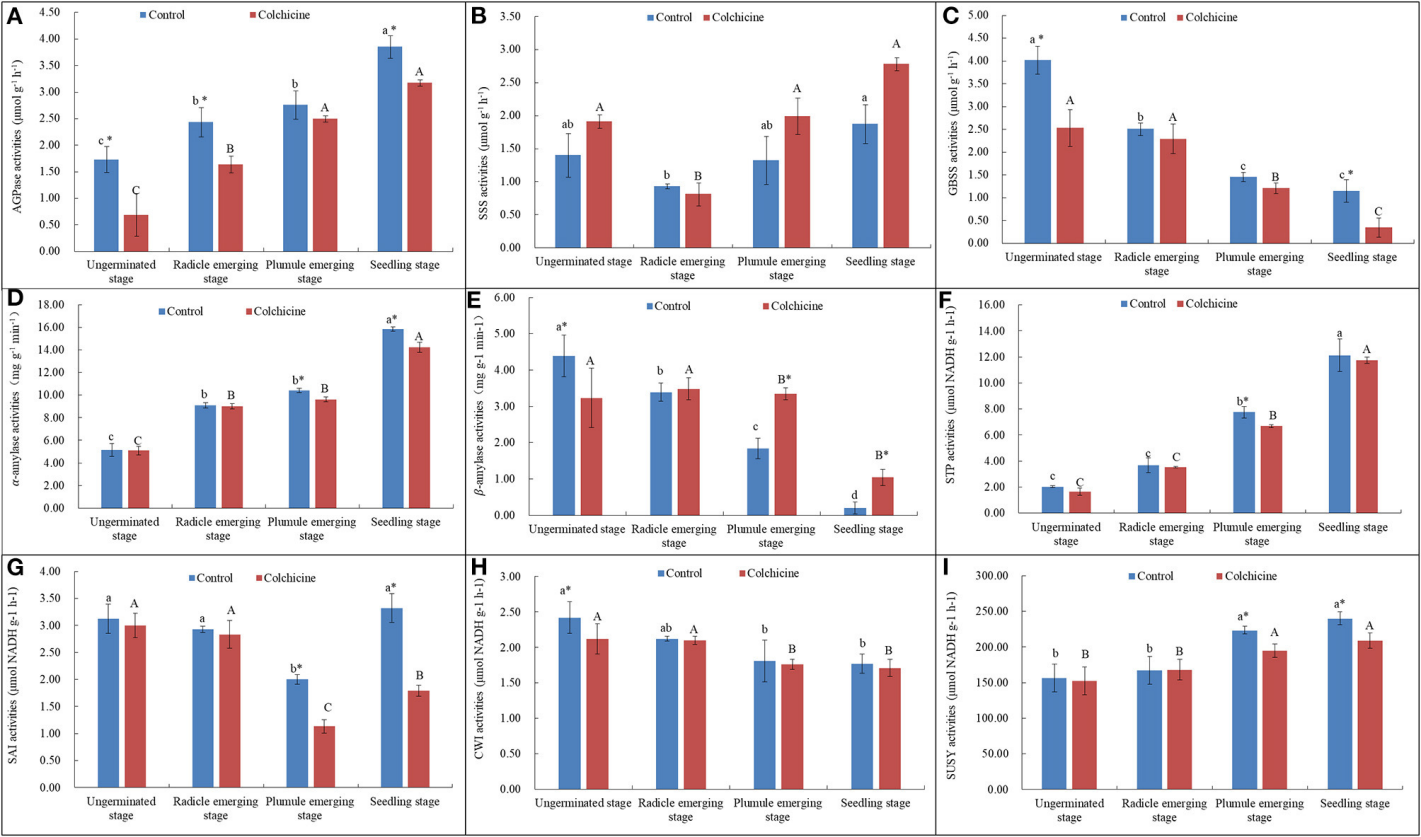
Enzymatic activities of the key carbohydrate-metabolizing enzymes during seed germination and the growth process in the media supplemented with and without 0.6% colchicine. **(A)** AGPase activities. **(B)** SSS activities. **(C)** GBSS activities. **(D)** α-amylase activities. **(E)** β-amylase activities. **(F)** STP activities. **(G)** SAI activities. **(H)** CWI activities. **(I)** SUSY activities. Different lowercase letters indicated significant difference between different developmental stages in the control, and different uppercase letters indicated significant difference between different developmental stages in the colchicine treatment at *P* < 0.05. *Indicated significant difference between the control and colchicine treatment.

The contents of endogenous starch and soluble sugars in seeds inoculated in the media supplemented without colchicine and with the highest concentration (0.6%) were determined and compared (Figure 4). The related key enzymatic activities were also measured and compared (Figure 5) to analyze the influences of colchicine on sugar metabolism in seeds during germination and seedling growth.

The endogenous starch contents decreased sharply in seeds when radicle emerged, and then constantly decreased during the following stages (Figure 4A). The starch content in seeds was not significantly different between the two kinds of media during the ungerminated and seedling stage but was significantly different during radicle and plumule emerging stages at *P* < 0.01 and 0.05 levels. The seeds inoculated in the media containing colchicine had higher starch contents at the radicle- and plumule-emerging stages.

As for the endogenous soluble sugar in seeds, their contents increased sharply during the radicle-emerging stage, and then increased slightly during the following stages in control but did not show an apparent trend in seeds inoculated in the media containing colchicine (Figure 4B). It could be concluded that the most endogenous starch stored in seeds was consumed during the radicle-emerging stage, and, meanwhile, colchicine significantly suppressed the starch consumption during the radicle and plumule stages. Hence, the endogenous soluble sugar contents were lower in seeds in the media containing colchicine than in those in the media without colchicine during the whole seed germination and seedling growth process. Especially in the seedling stage, the value of the endogenous soluble sugar means was significantly different at *p* < 0.05 levels.

### Dynamical Changes of Activities of Starch-Metabolizing Enzymes During Seed Germination and Seedling Growth

In the synthesis direction of starch, it was observed that AGPase activities (Figure 5A) increased constantly, while GBSS activities (Figure 5C) decreased significantly during seed germination and seedling growth. As for the activities of SSS, they showed a trend that decreased firstly at the radical emerging stage and then increased slightly and constantly during the following stages (Figure 5B). In the cleavage direction, it was observed that α-amylase (Figure 5D) and STP activities (Figure 5F) increased constantly, while β-amylase (Figure 5E) activities decreased constantly during the whole process of seed germination and seedling growth.

Generally, α-amylase and STP showed significantly higher activity values as compared to AGPase, SSS, GBSS, and β-amylase. This result indicates that starch degradation was one of the main physiological activities during seed germination and seedling growth, and α-amylase and STP played the key role in starch degradation as compared to β-amylase. In the media supplemented with colchicine, only the enzymatic activities of SSS and β-amylase were increased, but did not play an important role during seed germination and seedling growth due to their low activity values (Figures 5B,E). However, other enzymes, such as AGPase, GBSS, α-amylase, and STP showed lower values in the media containing colchicine (Figures 5A,C,D,F). Especially at the stage of seedling growth, the activities of AGPase, GBSS, and α-amylase were significantly suppressed by colchicine.

### Dynamical Changes of Activities of Sucrose-Catabolizing Enzymes During Seed Germination and Seedling Growth

Sucrose-catabolizing enzymes in seeds, including SAI, CWI, and SUSY, were determined for their activities during the process of germination and seedling growth (Figures 5G–I). SAI activities in seeds inoculated in the media without colchicine decreased significantly during seed germination but deeply increased at the seedling growth stage with the lowest values at the plumule-emerging stage (Figure 5G). While, in the media containing colchicine, a similar trend was also noticed that the SAI activities decreased firstly and then increased significantly with the lowest values at the plumule-emerging stage. However, the SAI activities in seeds were significantly lower in the media containing colchicine than in the media without colchicine at the plumule-emerging and seedling-growth stages. This result implied that the inhibiting effect of colchicine on SAI activities mainly occurred at the later stage of the seed germination and seedling growth process.

CWI activity in seeds decreased continuously with seed germination and seedling growth regardless of whether the media contained colchicine or not. This implies that CWI did not play an important role in seed germination and seedling growth (Figure 5H). Colchicine also decreased the CWI activities in seeds at all germination stages, but the differences were not significant.

In contrast to SAI and CWI, the activities of SUSY showed a continuously increasing trend with seed germination and seedling growth (Figure 5I). SUSY showed higher activity values as compared to SAI and CWI, implying that SUSY played a more important role during the process of seed germination and seedling growth. Additionally, colchicine significantly suppressed the SUSY activities at the plumule-emerging and seedling-growth stages, which was similar to the trend of SAI, α-amylase, and STP. Therefore, colchicine did not only affect the germination rates of *D. brandisii* seeds but also inhibited the subsequent seedling height growth by inhibiting the activities of sugar-metabolizing enzymes in seeds, which further inhibited the starch and sucrose degradation in seeds during seed germination and seedling growth.

### Correlation Analysis Between Seedling Height Growth and Sugar Metabolism in Media

**Figure 6:**
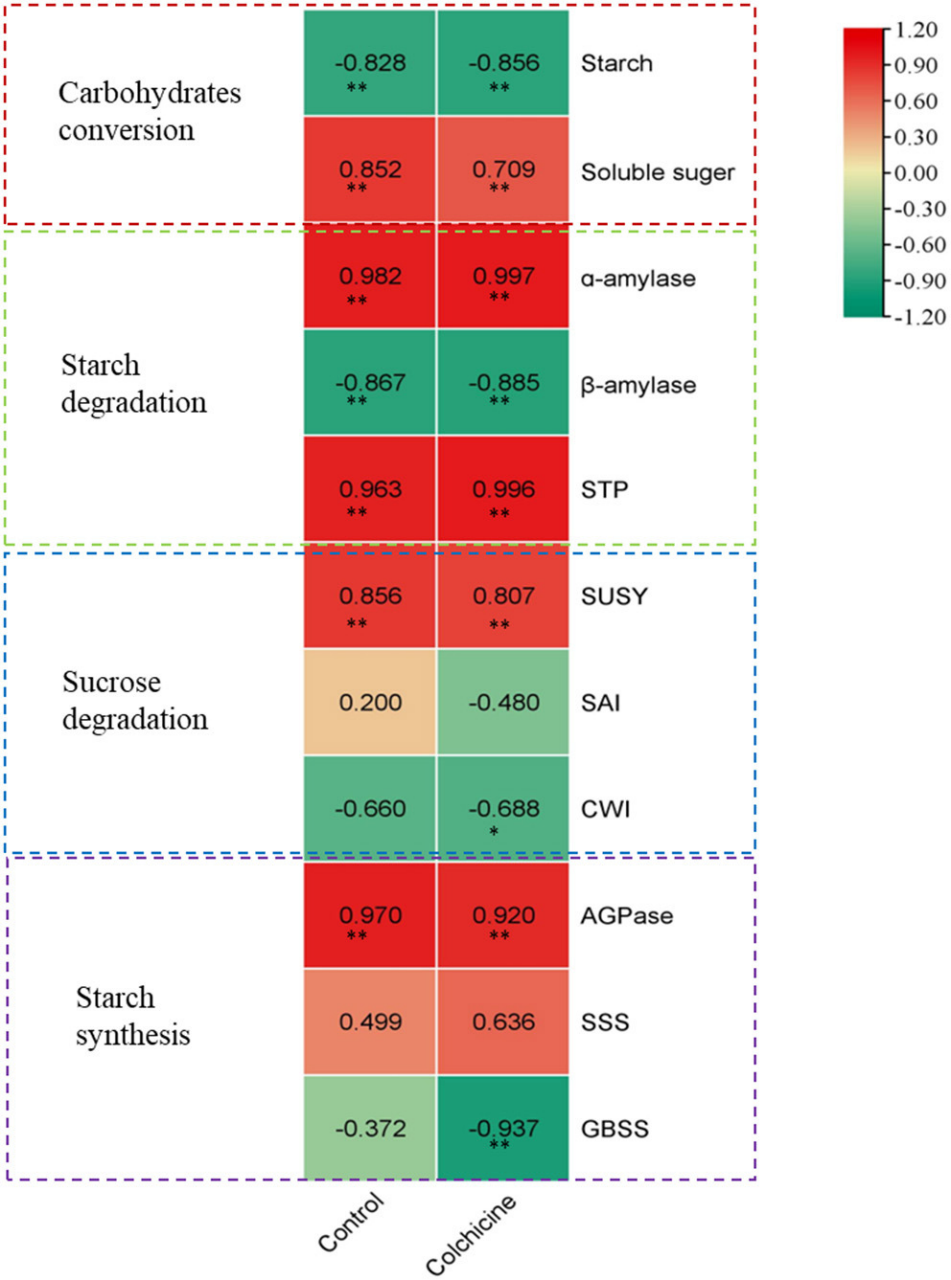
Correlation analysis between seedling height growth of *D. brandisii* and sugar metabolism. The darkness of the color indicates the ranking; the darkest red marked the highest value of positive correlation, and the darkest green marked the highest value of negative correlation.

By comparison, we noticed the height growth of seedlings during the whole germination stage coincided well with the dynamical changes of the contents of starch and soluble sugar, and the activities of sugar-metabolizing enzymes. Hence, a Pearson correlation analysis was performed between the seedling heights and the sugar metabolism at different germination stages (Figure 6). The correlation coefficients of the seeds in media containing colchicine and in media without colchicine were also compared.

Correlation analysis showed that the seedling height growth correlated significantly and negatively with a decrease of starch content in seeds but correlated significantly and positively with an increase of soluble sugar content at the *p* < 0.01 level. Seedling height growth also showed significant and positive correlations with α-amylase and STP activities but correlated significantly and negatively with β-amylase at the *p* < 0.01 level. For the seeds inoculated in media containing colchicine, the correlation showed a similar trend between seedling height growth and starch degradation. This result indicated that the seedling height growth of *D. brandisii* correlated significantly with the starch degradation, in which α-amylase and STP played a key role as compared to β-amylase. A similar trend was also shown in the correlation between seedling height growth and starch degradation in seeds inoculated in media containing colchicine. However, the correlation coefficients were different for the seeds inoculated in the media containing colchicine and containing no colchicine (Figure 6). Generally, the height growth of seedlings in media showed more negative and significant correlations (*p* < 0.01) with starch contents, but more positive and significant correlations (*p* < 0.01) with the activities of starch-catabolizing enzymes (α-amylase and STP) as compared to those of seedlings in media without colchicine. Additionally, the correlation coefficient between seedling height and soluble sugar content also decreased in the media containing colchicine.

In sucrose degradation, it was noticed that the height growth of seedlings correlated positively and significantly (*p* < 0.01) with SUSY activities in the media containing no colchicine, but did not show significant positive or negative correlations with SAI and CWI activities (Figure 6). Hence, SUSY played a more important role in seedling height growth as compared to SAI and CWI. While, for the seedlings in media containing colchicine, their height growth not only showed significant and positive correlations (*p* < 0.01) with SUSY activities but also showed significant and negative correlations (*p* < 0.05) with CWI, which also indicated that SUSY played the key role in sucrose degradation. Meanwhile, the correlation coefficients also showed a decrease for the seedlings in the media containing colchicine as compared to those of the control. It might be because colchicine decreased the SUSY activity in seedlings that the correlation coefficient also decreased. Generally, the seedling height growth of *D. brandisii* was closely related to the activities of α-amylase, STP, and SUSY during starch and sucrose degradation.

## Discussion

Bamboo plants seldom bloom and remain in a vegetative phase for decades or even a century, followed by flowering and death (Zheng et al., 2020). However, not all bamboos can produce seeds after flowering. Du et al. (2000) found that only 56% of bamboo species could bear seeds, according to their investigation on 68 bamboo flowering species. According to our observations on several bamboo species, bamboo seeds fall off easily from the spikelets once they mature. It is because bamboo seeds have been hardly gathered that only a few works have, so far, focused on the germination physiology of species such as *Dendrocalamus strictus* (Chand and Sood, 2008; Sarkar et al., 2020), *Phyllostachys edulis* (Shi et al., 2019; Emamverdian et al., 2021), *Chimonobambusa utilis* (Shi et al., 2019; Zhang et al., 2019), and *Neosinocalamus affinis* (Lin et al., 2019). Additionally, seeds of some other bamboo species, such as *D. asper, D. giganteus, D. membranaceus, D. minor, D. sinicus, D. yunnanicus, Schizostachyum funghomii, Chimonocalamus pallen, Qiongzhuea tumidissinosa*, and *Sasa veitchii* var. *hirsute*, have been gathered, and their related morphological characteristics determined and germination rates recorded (Devi et al., 2012; Xu et al., 2013; Yang et al., 2013; Abe and Shibata, 2014). However, systematic anatomical and physiological works are lacking. In the present study, seed germination and seedling growth of *D. brandisii*, as well as the related sugar metabolism, were determined and analyzed in detail. The physiological effects of colchicine on such seeds and seedlings were analyzed.

### Germination Characteristics of *D. brandisii* Seeds

Many plants hedge their reproductive “bets” through seed dormancy mechanisms that stagger germination or delay it until suitable conditions prevail (Rees, 1997; Bellairs et al., 2008). Seed dormancy has been demonstrated in several temperate-zone bamboos (Matumura and Nakajima, 1981; Taylor and Zisheng, 1988). Abe and Shibata (2014) reported that the seeds of *Sasa veitchii* var. *hirsuta* required a low-temperature environment to end dormancy for germination. Wang et al. (2020) also reported significant seed dormancy in *Fargesia qinlingensis*. As a tropical bamboo species, the seeds of *D. brandisii* germinated promptly once inoculated in the media and achieved their highest germination rates on the 4th day, implying no seed dormancy in *D. brandisii* seeds. This result also supported the inference or demonstration of other works that the seeds of tropical bamboo species lacked a seed dormancy mechanism (Janzen, 1976; Wong, 1981; Kondas, 1982; Venkatesh, 1984; Koshy and Harikumar, 2001; Rawat and Thapliyal, 2003; Bellairs et al., 2008). Bellairs et al. (2008) considered that the high and rapid germination of tropical bamboo seeds might be because the establishment of rhizomes and storage of nutrients during the seedling's first wet season demand maximum use of the wet season-growing period.

The seed germination period is different in different bamboo species according to the previous works. The seed germination period of *Oldeania alpine* was reported to be 8 days, with the germination starting on the 4th day after sowing (Zhang et al., 2016). Seed germination was reported to start on the 7th and completed on the 17th day in *Arundinaria alpina*, and started on the 4th day and completed on the 9th day in *Oxytenanthera abyssinica* (Bahru et al., 2015). Seeds of *D. brandisii* achieved their highest germination rates on the 4th day and finished their germination in 21 days. Therefore, seed germination time, germination period, and germination rates were different in different bamboo species.

### Germination and Seedling Height Growth and Inhibitory Effects of Colchicine

*D. brandisii* seeds inoculated in all media achieved their highest germination rates on the 4th day, implying colchicine did not affect their basic germination patterns but decreased the seed germination rate of each day. Total germination rates, germination potentials, and seedling survival rates also showed a continuous decreasing trend with colchicine contents in media, and the death rates increased constantly. Survival rates of seedlings were lower than their germination rates in the media containing high colchicine content, indicating that high content of colchicine caused the seedling death. Kwon et al. (2016) reported that a high concentration of colchicine significantly decreased seed germination of *Codonopsis lanceolata*. Another previous study also demonstrated that colchicine in high concentration had a negative effect on the germination of seeds, and survival plants recovered in the medical plant *Salvia hains* (Grouh et al., 2011). Bakry et al. (2007) and Sourour et al. (2014) reported the same finding on *Hordeum vulgare* and *Vicia narbonensis*, respectively. However, no works compared the inhibitory effects of colchicine on seed germination and on seedling growth.

No significant morphological differences were observed for the seedlings in the media containing colchicine as compared to the control, except for a few seeds in the media containing high colchicine content, which could not extend their radicle and plumule normally and finally died. According to the anatomical observations, it was noticed that the plumule of seeds in the media containing colchicine did not show a significant difference in anatomical structure as compared to those of the control, but their developmental levels were lower. Therefore, colchicine did not completely cause the aberrant development of *D. brandisii* seeds but significantly inhibited germination by retarding the development of the plumule. Moreover, seedling height growth was also significantly restrained by colchicine, and the inhibitory effects increased significantly with colchicine content in media. This might be because the high content of colchicine in media retarded tissue development and further inhibited the differentiation and development of radicle and plumule and further limited the subsequent height and diameter growth of seedlings. Ayu and Nurwahyuni (2019) also reported that high content of colchicine affected significantly plant height as well as dry and fresh weight. The comparison of the seedling height of *D. brandisii* at the 7th, 14th, 21st days with those of the control also revealed that the inhibitory effects of colchicine on height growth were more significant at the later stage than at the early stage during seedling height growth. Therefore, the high content of colchicine not only significantly inhibited germination and seedling growth but also caused its death during the later stage. Colchicine showed more severe inhibitory effects on seedling growth than on seed germination of *D. brandisii*. Similar findings were reported in other plant species where high mortality rates occurred with high concentrations of colchicine (Vichiato et al., 2014; Choopeng et al., 2019; Huy et al., 2019). The inverse relationship between colchicine concentrations and plant survival has been shown on some orchids (Petchang, 2010; Huy et al., 2019). Kazemi and Kaviani (2020) considered that mortality caused by colchicine was different for various species, mainly depending upon its concentrations, and a high concentration of colchicine was toxic for plant cells and could cause an imbalance between physiological and biochemical processes. An early study also reported that higher concentrations of colchicine could cause death by inhibiting DNA synthesis (Noodén, 1971).

### Physiology of Germination and Seedling Height Growth and an Inhibitory Mechanism of Colchicine

During germination and early seedling growth, the stored food reserved in the starchy endosperm is broken down by a variety of hydrolytic enzymes (Taiz and Zeiger, 2002). Starch is the major reserve material present in the endosperm (Brar et al., 2013). Carbohydrate metabolism is a fundamental biochemical process in plants that constantly supply energy for living cells, in which AGPase, SSS, and GBSS are related to starch synthesis, while α- and β-amylase and STP are related to starch degradation in plants (Wang et al., 2006). α- and β-amylase are the two main enzymes responsible for starch degradation during seed germination (Taiz and Zeiger, 2002). In our work, α-amylase and STP played a key role in starch degradation due to their significantly higher activity values and the increasing trend of activities during germination and seedling growth as compared to β-amylase in *D. brandisii* seeds. Additionally, the continuously increasing activities of AGPase and SSS revealed the starch synthetic capacity in seeds was increasing during germination and seedling growth under the adequate supply of sucrose in the medium. The sharp decrease in starch content and increase in soluble sugars in the seeds during the radicle-emerging stage revealed that starch degradation was a crucial physiological activity for seed germination.

SAI, CWI, and SUSY hydrolyze soluble sugars, and their substrates flow to the pathways of glycolysis and cellulose synthesis (Wang et al., 2006). Soluble acid invertase activities are usually high in rapidly growing tissues (Zhu et al., 1997) and are also involved in the sugar composition of sink tissues and osmoregulation and cell enlargement (Roitsch and González, 2004). SUSY-catalyzed metabolism was linked to biosynthetic processes of cell walls and storage products (Winter and Huber, 2000). In *D. brandisii* seeds, SAI activities firstly showed a decreasing trend during seed germination, and then significantly increased during seedling growth, implying that SAI began to function and play its role during seedling growth. CWI activities showed a continuously decreasing trend, while SUSY activities showed a constantly increasing trend during seed germination and seedling growth. In addition, SUSY showed a significantly higher activity value that was ~10-fold of the activities of SAI and CWI. Therefore, SUSY played a crucial role in seed germination and seedling growth.

As a widely used mitotic inhibitor for the induction of polyploidy in plants (Manzoor et al., 2019), most works have focused on the inhibitory effects of colchicine on cell division and enlargement. However, its physiological effects on carbohydrate metabolism during seed germination and seedling growth have been neglected. Moreover, starch degradation is a fundamental activity during the germination and seedling growth of starchy seeds. In the present study, the seeds showed significantly higher starch content but lower soluble sugar content in the media containing colchicine than those in the control during seed germination and seedling growth, which indicated that starch degradation was inhibited by colchicine. The lower α-amylase and STP activities in seeds inoculated in the media containing colchicine also supported this conclusion.

Colchicine caused significant growth inhibition during the seedling growth stage, and the inhibition was not obvious at the germination stage, which was mainly because seedling growth needed more energy from sugar metabolism as compared to the germination stage. In this study, we noticed that colchicine significantly inhibited the activities of α-amylase, STP, SAI, and SUSY enzymes at the plumule-emerging stage and the seedling growth stage, which affected the carbohydrate degradation and further inhibited the seedling growth. SAI activities were linked with cell enlargement, and SUSY activities were linked with the biosynthesis of the cell wall (Winter and Huber, 2000; Roitsch and González, 2004). Therefore, colchicine inhibited seedling growth because not only it inhibited cell division and DNA synthesis (Noodén, 1971; Manzoor et al., 2019) but also because colchicine inhibited sucrose degradation, which further inhibited cell enlargement and cell wall synthesis.

Correlation analysis revealed that the seedling height growth not only correlated significantly and positively with the starch degradation in seeds by increasing the activities of α-amylase and STP but also correlated significantly and positively with the sucrose degradation by increasing the SUSY activities. However, SAI and CWI did not show significant correlations with seedling growth. This result was different from that of the young shoot elongation of *Fargesia yunnanensis*, during which SAI played an important role (Wang et al., 2006). In a medium containing colchicine, starch degradation was inhibited, and the seedling height growth showed higher correlations with the decrease of starch content and the increase of α-amylase and STP activities in seeds. This result revealed that seed germination and seedling growth relied more heavily on endogenous starch degradation under colchicine supplement conditions. On the other hand, colchicine might decrease the absorption capacity of exogenous sugars in seeds.

Contrary to starch degradation, the correlation between seedling height and SUSY activities decreased in the media containing colchicine as compared to those in the media without colchicine, which indicated that colchicine showed more negative effects on sucrose degradation than on starch degradation. In the media containing colchicine, the decrease of correlation coefficients between seedling height growth and soluble sugar content also revealed that colchicine suppressed the sucrose degradation in seeds, which further inhibited the seedling height growth. Additionally, it was noticed that the starch content still decreased significantly during germination and seedling growth, although the seeds were inoculated in the media supplemented with sufficient sucrose. This implied that endogenous starch stored in seeds was still the main source of energy for seed germination and seedling growth under tissue culture conditions.

## Conclusion

Seeds of *D. brandisii* are nut-like caryopsis and spherical. In this work, they achieved their highest germination rate on the 4th day and finished the whole germination in 21 days. The germination rates constantly decreased with colchicine content in media, but the germination pattern did not change. A high concentration of colchicine not only increased the death rates of seeds, inhibited the germination rates and germination potentials, but also decreased the later seedling survival rates and height growth. Seed germination and seedling growth correlated significantly with the endogenous starch and soluble sugar degradation, in which α-amylase, STP, and SUSY played the key role. Colchicine inhibited seed germination and seedling growth by suppressing the α-amylase, STP, and SUSY activities. Colchicine showed more negative effects on sucrose degradation than on starch degradation. Under tissue culture, seed germination and seedling growth still relied on endogenous starch degradation.

## Data Availability Statement

The original contributions presented in the study are included in the article/supplementary material, further inquiries can be directed to the corresponding author/s.

## Author Contributions

ZL and FZ analyzed the data and performed most of the experiments, analyzed the data, and participated in interpretation. DJ and YW collected and processed the samples, analyzed the data, and participated in interpretation. SW designed the project, provided supervision, and took part in writing the manuscript. All authors have read and approved the final version of the manuscript.

## Funding

This manuscript was fully funded by the National Key R&D Program of China (2021YFD2200500) and the National Natural Science Fund of China (31560196).

## Conflict of Interest

The authors declare that the research was conducted in the absence of any commercial or financial relationships that could be construed as a potential conflict of interest.

## Publisher's Note

All claims expressed in this article are solely those of the authors and do not necessarily represent those of their affiliated organizations, or those of the publisher, the editors and the reviewers. Any product that may be evaluated in this article, or claim that may be made by its manufacturer, is not guaranteed or endorsed by the publisher.
